# Nano-scale machining of polycrystalline coppers - effects of grain size and machining parameters

**DOI:** 10.1186/1556-276X-8-500

**Published:** 2013-11-22

**Authors:** Jing Shi, Yachao Wang, Xiaoping Yang

**Affiliations:** 1 Department of Industrial and Manufacturing Engineering, North Dakota State University, Fargo, ND 58108, USA; 2 EBU Global Manufacturing Engineering Department, Cummins Inc., Columbus, IN 47202, USA

**Keywords:** Nano-scale machining, Grain size, Molecular dynamics simulation, Inverse Hall–Petch relation, Cutting forces, Grain boundary

## Abstract

In this study, a comprehensive investigation on nano-scale machining of polycrystalline copper structures is carried out by molecular dynamics (MD) simulation. Simulation cases are constructed to study the impacts of grain size, as well as various machining parameters. Six polycrystalline copper structures are produced, which have the corresponding equivalent grain sizes of 5.32, 6.70, 8.44, 13.40, 14.75, and 16.88 nm, respectively. Three levels of depth of cut, machining speed, and tool rake angle are also considered. The results show that greater cutting forces are required in nano-scale polycrystalline machining with the increase of depth of cut, machining speed, and the use of the negative tool rake angles. The distributions of equivalent stress are consistent with the cutting force trends. Moreover, it is discovered that in the grain size range of 5.32 to 14.75 nm, the cutting forces and equivalent stress increase with the increase of grain size for the nano-structured copper, while the trends reserve after the grain size becomes even higher. This discovery confirms the existence of both the regular Hall–Petch relation and the inverse Hall–Petch relation in polycrystalline machining, and the existence of a threshold grain size allows one of the two relations to become dominant. The dislocation-grain boundary interaction shows that the resistance of the grain boundary to dislocation movement is the fundamental mechanism of the Hall–Petch relation, while grain boundary diffusion and movement is the reason of the inverse Hall–Petch relation.

## Background

Built on the classical Newton's Second Law, molecular dynamics (MD) simulation has been proven to be an effective tool to study many underlying intriguing mechanisms of material processing. This technique works particularly well with very small scales, which could be often ineffective for any experimental approaches or other mainstream numerical simulation approaches. As such, it has been applied to tackle countless interesting problems in the area of material processing, including the formation of dislocation, development of fracture, evolution of friction and wear, and effects of processing parameters in various processes. Nano-scale machining is one of those processes, and it is an important method to create miniaturized components and features. A substantial amount of research has been carried out on nano-scale machining by MD simulation. The pioneer works of Inamura et al. [1,2] adopted this technique to investigate the mechanics, energy dissipation, and shear deformation in nano-scale machining of monocrystal copper. It was argued that the theory of continuum mechanics is not applicable to nano-scale machining. Meanwhile, the deformation mechanism in the primary shear zone seems to be related to buckling due to severe compression in that area, while the deformation at the secondary shear zone appears to be the result of shear plastic deformation by yield shear stress.

Numerous other studies on MD simulation of nano-scale machining have emerged since 1990s. Ikawa et al. [3] investigated the minimum thickness of cut (MTC) for ultrahigh machining accuracy. It was discovered that an undercut layer of 1 nm is achievable for machining of monocrystal copper with a diamond tool. Fang and Weng [4] also simulated nano-scale machining of monocrystal copper using a diamond tool by focusing on friction. It was found that the calculated coefficients of friction in nano-scale machining are close to the values obtained in macro-scale machining. Shimada et al. [5,6] adopted MD simulation to analyze 2D machining of monocrystal copper using diamond tools. It was found that disordered copper atoms due to tool/material interaction can be self re-arranged after the cutting edge passes the affected area. For simulating nano-scale machining of monocrystal copper, Ye et al. employed the embedded atom method (EAM) to model the potential energy of copper atoms [7]. Compared with other potential energy models for nano-scale machining, the EAM potential can produce comparable results, and thus, it is regarded as a viable alternative. Komanduri et al. [8,9] conducted extensive simulation works on nano-scale machining of monocrystal aluminum and silicon. The works reveal the effects of various parameters, such as cutting speed, depth of cut, width of cut, crystal orientation, and rake angle, on chip formation and cutting force development. The effort on investigating the effects of machining parameters on the performances of nano-scale machining has never stopped. For instance, Promyoo et al. [10] investigated the effects of tool rake angle and depth of cut in nano-scale machining of monocrystal copper. It was discovered that the ratio of thrust force to tangential cutting force decreases with the increase of rake angle, but it hardly changes with the depth of cut. Shi et al. [11] developed a realistic geometric configuration of three-dimensional (3D) single-point turning process of monocrystal copper and simulated the creation of a machined surface based on multiple groove cutting. A variety of machining parameters were included in this realistic 3D simulation setting. Meanwhile, other phenomena in nano-scale machining are also investigated by MD simulation approach. Tool wear appears to be one of the most studied topics. Zhang and Tanaka [12] confirmed the existence of four regimes of deformation in machining at atomistic scale, namely, no-wear regime, adhering regime, ploughing regime, and cutting regime. It was found that a smaller tip radius or a smaller sliding speed brings a greater no-wear regime. Cheng et al. [13] discovered that the wear of a diamond tool is affected by the cutting temperature as heat generation decreases the cohesive energy between carbon atoms. Another study of nano-machining revealed that the iron workpiece has anisotropic influence on diamond cutting tool graphitization, an important indicator of tool wear [14]. In addition, there are a number of studies in the literature on the brittle-ductile transition phenomenon of silicon material in nano-scale machining or indentation. For instance, Tanaka et al. observed amorphous phase transformation of silicon in nano-machining and that stable shearing of the amorphous region is necessary for ductile-mode machining [15]. Also, a numerical study of surface residual stress distribution of silicon during nano-machining process is presented by Wang et al. [16]. Their MD simulation results revealed that higher hydrostatic pressure beneath the tool rake face induces more drastic phase transformation and thus generates more compressive surface residual stress. MD simulation is also capable of modeling chip formation, separation, and evolution mechanism. For instance, Ji et al. [17] studied the tool-chip stress distribution in nano-machining of copper, and the results were compared to the existing models of conventional machining. Lin and Huang [18] studied nano-cutting process by MD simulation and proposed the innovative ‘combined Morse potential function and rigid tool space restrictions criterion’ as the chip separation criterion. It was used to establish the shape function of the FEM-MD combined model.

Existing studies on MD simulation of nano-scale machining usually adopt defect-free monocrystalline structures as the work material [19]. The most popular ones have been monocrystal copper, aluminum, and silicon. Nevertheless, the vast majority of engineering materials exist in polycrystalline (instead of monocrystalline) forms. It is not difficult to understand that machining polycrystalline structures may yield different results compared with machining monocrystalline structures. Moreover, the grain size in polycrystalline structures is often a controlling factor for material properties and material responses to deformation. It is important to investigate how it impacts the machining performance at nano/atomistic scale. In a preliminary study, Shi and Verma [20] constructed one polycrystalline copper structure, simulated nano-scale machining of the structure, and made a comparison with monocrystalline machining. It was discovered that for all cutting conditions simulated, the polycrystalline structure requires smaller cutting forces compared with the monocrystalline structure. This result might be expected as the existence of grain boundary is usually regarded as defects, and thus, it reduces material strength. However, many more interesting questions arise from the preliminary finding, such as ‘Will the polycrystalline structures with different grain sizes behave differently in nano-scale machining?’, ‘What are the roles of grain boundaries in measured machining performances?’, and ‘How do the machining parameters affect the performances of nano-scale polycrystalline machining?’ In an effort to answer these research questions, we carry out this study.

The effect of grain size and grain boundary on the material's mechanical property has been well discussed. Usually, the well-known Hall–Petch relationship is widely accepted. This relationship indicates that material strength increases with the decrease of grain size. However, for very fine nano-structured materials, this relationship may no longer hold. Yang and Vehoff [21] investigated the dependency of hardness upon grain size in nano-indentation experiments. With the indentation depth of less than 100 nm, it is clearly revealed that the local interaction between dislocations and grain boundaries causes various hardness dependences on indentation depth. Zhang et al. [22] carried out nano-indentation experiments on copper with grain sizes from 10 to 200 nm. It was found that at short dwell times, the hardness increases significantly with decreasing grain size. However, the difference substantially diminishes at longer times due to the rapid grain growth under the indenter. Similar reverse proportion relations between grain size and hardness are observed in indentation experiments at micro-scale in the literature. Li and Reece [23] discovered that grain size has a significant effect on surface fatigue behavior, and increasing grain size reduces the threshold for crack nucleation. Also, Lim and Chaudhri [24] showed that in the grain size range of 15 to 520 μm, the initial higher dislocation density for smaller grains is believed to cause higher Vickers hardness. More importantly, the rapid advance of numerical simulation techniques has enabled more detailed analysis of dislocations and grain boundaries in deformation of polycrystallines. For instance, with the help of MD simulation, the interaction of dislocations with a ∑ = 5(210)[001] grain boundary is analyzed, and the transmission of dislocation across the grain boundary is observed [25]. Another MD simulation study indicates that compared to bulk diamond crystal, substitution energies are found to be significantly lower for grain boundaries [26].

The remainder of the paper is organized as follows. In the next section, the MD model construction for nano-scale machining of polycrystalline is briefly introduced. The machining conditions for the simulation cases are also summarized. Thereafter, the simulation results of nano-scale machining are presented, in which the major observations are made regarding the effects of grain size and machining parameters. More importantly, a detailed discussion on the grain size effect is provided to reveal the governing mechanism in nano-scale machining. Finally, conclusions are drawn and future research is pointed out in the last section.

## Methods

### Simulation model construction

**Figure 1:**
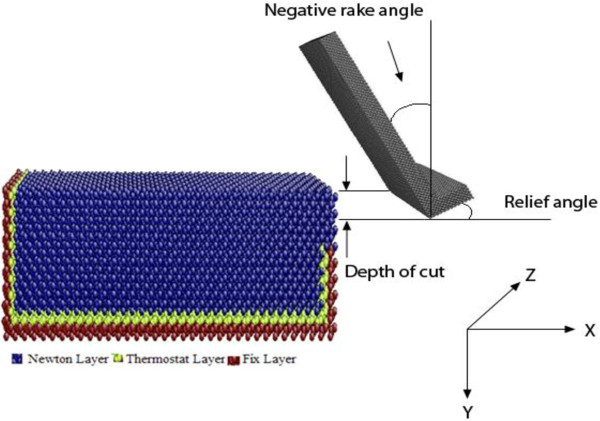
MD simulation model of nano-scale machining.

Figure 1 shows the overall MD simulation model constructed according to a 3D orthogonal machining configuration. For all the cases, the tool material is always diamond, and the work materials are polycrystalline coppers except for the benchmark case of monocrystalline copper. The diamond tool is oriented to achieve a rake angle of -30° and a relief angle of 30°, and it is treated as a rigid body in MD simulation. It can also be seen from Figure 1 that the work material atoms are categorized into three types - namely, fixed layer, thermostat layer, and Newton layer. The atoms in the fixed layer have fixed positions and only interact with the other two types of work material atoms. The thermostat layer lies between the fixed layer and the Newton layer. The atoms in the thermostat layer are used to stabilize the temperature of the system. For all the simulation cases, the copper workpieces have the identical dimension of 432 × 216 × 216 Å^3^. The polycrystalline copper structures are built based on the operation of Voronoi site-rotation and cut [27]. The simulation is carried out using LAMMPS, a general-purpose molecular dynamics simulation code developed by Sandia National Lab [28]. Post-processing codes are developed in-house to calculate machining forces, stress distributions, and dislocation development.

### Simulated machining cases and machining parameters

A total of 13 simulation cases are constructed to investigate (1) the effects of machining parameters in polycrystalline machining and (2) the effect of grain size of polycrystalline copper on machining performances. Table 1 summarizes the machining conditions for all the 13 cases. For the first task, we select three levels of machining speed, i.e., 25, 100, and 400 m/s; three levels of depth of cut, i.e., 10, 15, and 20 Å; and three levels of tool rake angle, i.e., -30°, 0°, and +30°. As such, the group of cases C4, C8, and C9 can be used to investigate the machining speed effect since the only different parameter among the three cases is the machining speed. For the same reason, the group of cases C4, C10, and C11 can be used to reveal how the depth of cut affects polycrystalline machining, and cases C4, C12, and C13 can be compared to show the effect of tool rake angle. Note that the lowest machining speed employed in this study is 25 m/s, which is still high even compared with the typical machining speeds (e.g., 5 to 10 m/s) of high speed machining. However, this arrangement is necessary because MD simulation is extremely computation intensive. For instance, the average computation time for a case with 400 m/s machining speed in this study is about 8 days on an Intel Core i7 3.2-GHz PC.

**Table 1:** Machining conditions for the 13 simulation cases of nano-scale machining

**Case number**	**Depth of cut (Å)**	**Tool rake angle (deg)**	**Machining speed (m/s)**	**Grain size (nm)**
C1	15	-30	400	Monocrystal
C2	15	-30	400	16.88
C3	15	-30	400	14.75
C4	15	-30	400	13.40
C5	15	-30	400	8.44
C6	15	-30	400	6.70
C7	15	-30	400	5.32
C8	15	-30	100	13.40
C9	15	-30	25	13.40
C10	10	-30	400	13.40
C11	20	-30	400	13.40
C12	15	0	400	13.40
C13	15	30	400	13.40

For the second task, the machining conditions are fixed, and six levels of grain size are created. The six grain sizes are 5.32, 6.70, 8.44, 13.40, 14.75, and 16.88 nm. They correspond to 256, 128, 64, 16, 12, and 8 face-centered cubic (fcc) grains within an identical work dimension and represent simulation cases C2 to C7, respectively. The comparison among the six cases can illustrate the effect of grain size on polycrystalline machining. To make the comparison complete, a monocrystalline copper structure is also created and simulated, which is represented by case C1.

### Potential formulations

The interaction between the copper atoms in the work material and the carbon atoms in the diamond tool can be modeled using the pairwise Morse potential [29]:

(1) $$U=D\left\{\text{exp}\left[-2\alpha \left(r_{\mathrm{ij}}-r_{0}\right)\right]-2\;\text{exp}\left[-\alpha \left(r_{\mathrm{ij}}-r_{0}\right)\right]\right\},$$

where *D* is the cohesion energy, *α* is a constant parameter, *r*_*ij*_ is the distance between the two atoms, and *r*_0_ is the distance at equilibrium. The parameters for the Morse potential between copper and carbon atoms are presented in Table 2.

**Table 2:** **Morse potential parameters for Cu-C interaction **[1]**,**[31]

**Parameter**	**Value**
*D* (eV)	0.1063
*α* (Å^-1^)	1.8071
*r*_0_ (Å)	2.3386
Potential cutoff distance (Å)	6.5

The interaction forces between copper atoms are modeled using the EAM potential, which is a multi-body potential energy function in the following form [30]:

(2) $$U=F_{\alpha }\sum_{j\neq i}\rho_{i}\left(R_{i,j}\right)+\frac{1}{2}\sum_{j\neq i}\varphi_{\alpha ,\beta }\left(R_{i,j}\right),$$

where the total energy (*U*) on atom *i* is the sum of the embedding energy *F* and the short-range pair potential energy *φ*, *ρ* is the electron density, and *α* and *β* are the element types of atoms *i* and *j*. The embedding energy is the energy to put atom *i* in a host electron density (*ρ*_*i*_) at the site of that atom. The pair potential term (*φ*) describes the electrostatic contributions. The EAM potential parameters are presented in Table 3.

**Table 3:** **EAM potential parameters for Cu-Cu interaction **[4]**,**[20]

**Parameter**	**Value**
Lattice constant (Å)	3.62
Cohesive energy (eV)	-3.49
Bulk modulus (GPa)	137
*C'* (GPa)	23.7
*C*_44_ (GPa)	73.1
Δ(*E*_bcc_ - *E*_fcc_) (meV)	42.7
Δ(*E*_hcc_ - *E*_fcc_) (meV)	444.8
Stacking fault energy (mJ/m^2^)	39.5
Vacancy: *E*_f_ (eV)	1.21

To calculate the cutting force, the individual interaction force on atom *i* due to atom *j* should be computed first by differentiating the potential energy. For each tool atom, the reaction forces should also be summed among its neighbor atoms. Then, the cutting force in vector form can be obtained by summing all the interaction forces on the cutting tool atoms:

(3) $$F=\sum_{i=1}^{N_{T}}\sum_{j}\frac{\partial U\left(r_{\mathrm{ij}}\right)}{\partial r_{\mathrm{ij}}},$$

where *F* is the cutting force and *N*_*T*_ is the number of atom in the cutting tool.

For the calculation of stress components *s*_*xx*_, *s*_*yy*_, *s*_*zz*_, *s*_*xy*_, *s*_*xz*_, and *s*_*yz*_ of atom *i*, the following equation is used:

(4) $$\chi =\frac{1}{\Omega }\sum_{i}^{N}\left(m_{i}v_{i}⊗v_{i}+\frac{1}{2}\sum_{i\neq j}r_{\mathrm{ij}}⊗\frac{\partial U\left(r_{\mathrm{ij}}\right)}{\partial r_{\mathrm{ij}}}\right),$$

where *χ* is the average virial stress component, Ω is the volume of the cutoff domain, *m*_*i*_ is the mass, *v*_*i*_ is the velocity of atom *i*, ⊗ denotes the tensor product of two vectors, and *N* is the total number atoms in the domain. To calculate the equivalent stress (*S*), the virial stress components are used:

(5) $$S=\sqrt{3\left(s_{\mathrm{xy}}^{2}+s_{\mathrm{yz}}^{2}+s_{\mathrm{xz}}^{2}\right)+\frac{1}{2}\left[{\left(s_{\mathrm{xx}}-s_{\mathrm{yy}}\right)}^{2}+{\left(s_{\mathrm{xx}}-s_{\mathrm{zz}}\right)}^{2}+{\left(s_{\mathrm{zz}}-s_{\mathrm{yy}}\right)}^{2}\right]}.$$

## Results and discussion

### Effect of machining parameters

#### ***Effect of depth of cut***

**Figure 2:**
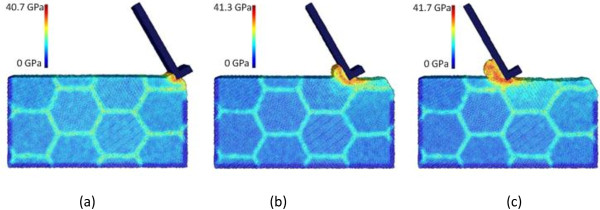
**Chip formations and equivalent stress distributions in nano-scale polycrystalline machining for case C10.** At the tool travel distances of **(a)** 30, **(b)** 120, and **(c)** 240 Å.

**Figure 3:**
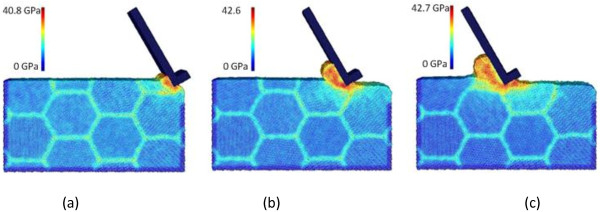
**Chip formations and equivalent stress distributions in nano-scale polycrystalline machining for case C4.** At the tool travel distances of **(a)** 30, **(b)** 120, and **(c)** 240 Å.

**Figure 4:**
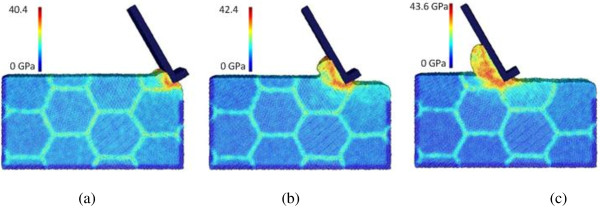
**Chip formations and equivalent stress distributions in nano-scale polycrystalline machining for case C11.** At the tool travel distances of **(a)** 30, **(b)** 120, and **(c)** 240 Å.

As mentioned above, cases C10, C4, and C11 adopt three levels of depth of cut, namely, 10, 15, and 20 Å, respectively, while the other machining parameters are the same. For each case, three snapshots of machining progress at the tool travel distances of 30, 120, and 240 Å are presented. The results for the three cases are shown in Figures 2, 3, and 4, respectively. First of all, chip formation progress can be observed here. For all the three cases, the machined chip accumulates in front of the tool rake face as the tool advances. The chip volume is approximately proportional to the depth of cut. However, the cutting chip thicknesses for cases C10, C4, and C11 are measured to be 18, 40, and 45 Å, respectively. The increase of chip thickness is more significant when the depth of cut increases from 10 to 15 Å, compared with the increase period from 15 to 20 Å.

Figures 2, 3, and 4 also provide the information of equivalent stress distribution in polycrystalline machining. It can be found that the stress distribution pattern of nano-scale polycrystalline machining is overall consistent with that of conventional machining, as well as that of nano-scale machining of monocrystalline structures [20,31]. For all the cases, the stress concentration is observed in the primary shear zone, where the chip is formed by high-strain-rate shearing in the primary shear zone, as well as the second shear zone, which is the friction-affected zone between the tool rake face and the chip. For each case, the maximum stress occurs at the primary shear zone and it increases as the depth of cut increases. For instance, at the tool travel distance of 240 Å, the maximum equivalent stress values are 41.7, 42.7, and 43.6 GPa for cases C10, C4, and C11, respectively. Meanwhile, our results indicate that the equivalent stress on grain boundaries is generally 30% to 60% higher than the stress inside the grains. Note that the difference of equivalent stresses on grain boundaries and inside the grains is not only caused by the exertion of cutting force. It is believed that the crystallographic orientation of grains could introduce stress concentration on and nearby boundaries. The literature also indicates that a higher amount of stress and lattice distortion can develop nearby the grain boundaries [32].

In addition, no crack is observed during the entire machining process for all cases. This is a reasonable result based on the MD simulation study by Heino et al. [33], in which the crack initiation and propagation mechanism within a copper material is investigated. It was estimated that the critical tensile stress for crack initiation is around 15 GPa. However, in our simulation, the maximum tensile stress of the as-machined surface in the vicinity of the cutting tool is around 3 GPa, which is much smaller than the critical crack initiation tensile stress. In addition, the use of a negative rake angle also helps avoid cracks and improve machined surface quality in nano-machining process [16].

**Figure 5:**
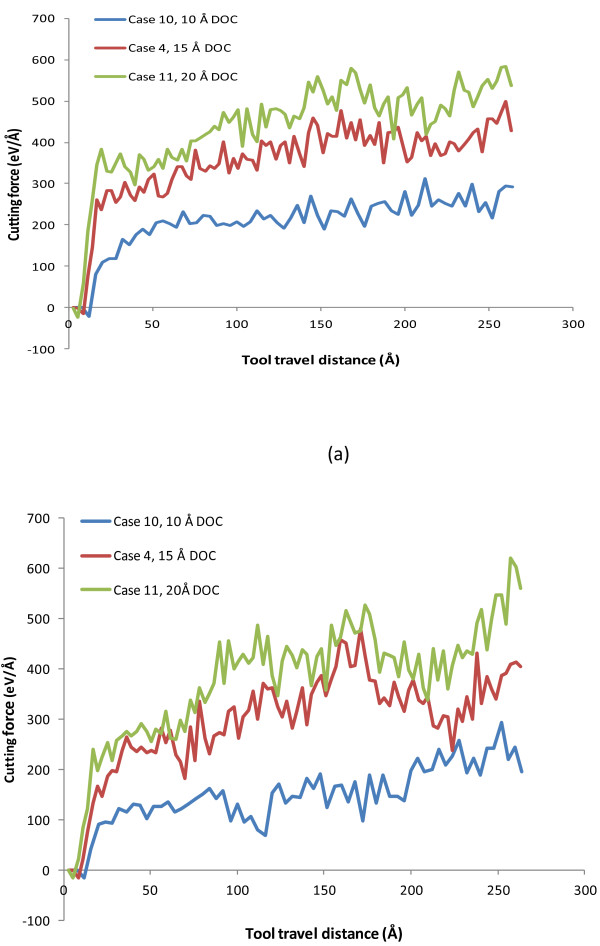
**Evolution of cutting forces for three cases with three depths of cut (DOC). (a)** Tangential force, *F*_*x*_ and **(b)** thrust force, *F*_*y*_.

Figure 5a,b compares the evolution curves of cutting force components, *F*_*x*_ and *F*_*y*_, for cases C10, C4, and C11. *F*_*x*_ and *F*_*y*_ are the force components along the *X* and *Y* axes as indicated in Figure 1, and they represent the tangential force and the thrust force, respectively. It can be seen that for all the cases, both *F*_*x*_ and *F*_*y*_ increase rapidly at the beginning of machining process, but the trend of increase slows down after the tool travel distance is beyond about 30 Å. Overall, both the tangential and thrust forces increase with the increase of depth of cut. Nevertheless, a more significant increase in both force components is observed as the depth of cut increases from 10 to 15 Å, compared with that when the depth of cut increases from 15 to 20 Å.

Meanwhile, to make a direct and fair comparison, the average *F*_*x*_ and *F*_*y*_ values are obtained by averaging the fluctuating force values obtained during the travel distance period of 160 to 280 Å, which represents the relative stable stage of the entire machining process. The results are summarized in Table 4. As the depth of cut increases from 10 to 15, and then to 20 Å, the tangential force increases from 254.41 to 412.16, and then to 425.32 eV/Å, and the thrust force increases from 199.99 to 353.59, and then to 407.26 eV/Å, respectively. The increase of cutting force due to the increase of depth of cut in nano-scale polycrystalline machining should not be a surprise. More energy is needed to remove more material, and this actually applies to the machining process at all length scales [10,31,34]. Moreover, the ratios of tangential force to thrust force, *F*_*x*_/*F*_*y*_, for the three cases are calculated. It is found that *F*_*x*_/*F*_*y*_ decreases as the depth of cut increases. This means that as the depth of cut increases, the increase of thrust force is more significant than the increase of tangential force.

**Table 4:** Average cutting force values with respect to depth of cut

**Case number**	**Depth of cut (Å)**	***F***_***x ***_**(eV/Å)**	***F***_***y ***_**(eV/Å)**	***F***_***x***_**/*F***_***y***_
C10	10	254.41	199.99	1.27
C4	15	412.16	353.59	1.17
C11	20	509.94	454.92	1.12

#### ***Effect of tool rake angle***

**Figure 6:**
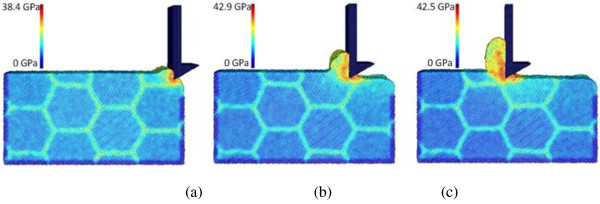
**Chip formations and equivalent stress distributions in nano-scale polycrystalline machining for case C12.** At the tool travel distances of **(a)** 30, **(b)** 120, and **(c)** 240 Å.

**Figure 7:**
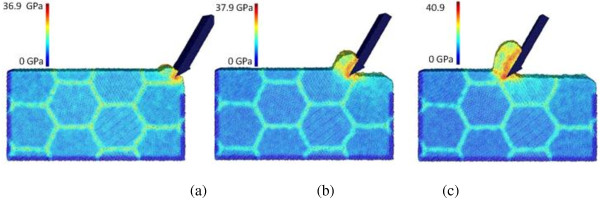
**Chip formations and equivalent stress distributions in nano-scale polycrystalline machining for case C13.** At the tool travel distances of **(a)** 30, **(b)** 120, and **(c)** 240 Å.

For this purpose, cases C4, C12, and C13 are compared because they adopt three different tool rake angles of -30°, 0°, and +30°, respectively. Figure 3 already shows the machining snapshots for case C4. Figures 6 and 7 illustrate the machining snapshots taken at the same tool travel distances for cases C12 and C13, respectively. Certainly, the rake angle dictates the chip formation/flow direction, and also, the chip geometries are somehow different among the three cases. By examining the equivalent stress distributions in the affected zones, it can be found that the primary shear zone becomes more distinguishable from the secondary shear zone when the rake angle changes from negative to positive. Also, the affected uncut zone ahead of the cutting tool becomes shallower when the rake angle changes from negative to positive. This indicates the severity of compression effect in the affected uncut zone.

**Figure 8:**
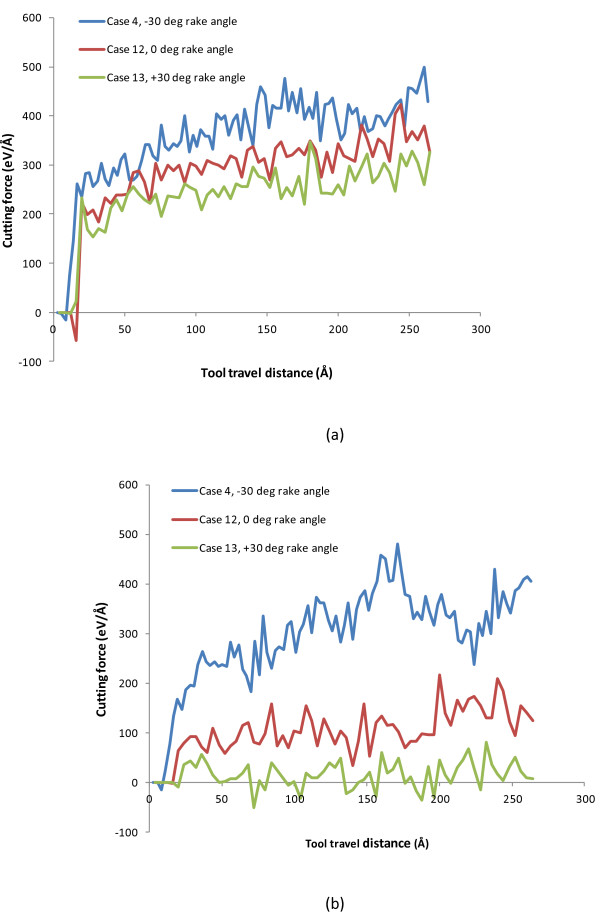
**Evolution of cutting forces for three cases with three rake angles. (a)** Tangential force, *F*_*x*_ and **(b)** thrust force, *F*_*y*_*.*

Similarly, the cutting force evolutions are compared to illustrate the effect of tool rake angle. As shown in Figure 8a,b, as the tool rake angle changes from -30° to 0°, and then to +30°, both the tangential force *F*_*x*_ and the thrust force *F*_*y*_ decrease and the deduction of thrust force is more pronounced. The average *F*_*x*_ and *F*_*y*_ values are also calculated to make a more direct comparison. As shown in Table 5, with the -30°, 0°, and +30° tool rake angles, the average tangential forces are 412.16, 338.73, and 280.80 eV/Å, respectively, and the thrust force values are 353.59, 132.68, and 19.43 eV/Å, respectively. The ratio of tangential force to thrust force, *F*_*x*_/*F*_*y*_, increases from 1.17 to 14.45 as the rake angle changes from -30° to +30°. Clearly, the more drastic compression effect between tool and workpiece induced by the negative rake angle causes much higher thrust force compared to the cases with zero or positive tool rake angle. As the rake angle becomes more negative, the thrust force needs to increase more significantly compared to the tangential force to overcome the plastic deformation resistance of the work material under the tool tip. This result is consistent with the literature on conventional machining and nano-scale monocrystalline machining [35,36].

**Table 5:** Average cutting force values with respect to tool rake angle

**Case number**	**Tool rake angle (deg)**	***F***_***x ***_**(eV/Å)**	***F***_***y ***_**(eV/Å)**	***F***_***x***_**/*F***_***y***_
C4	-30	412.16	353.59	1.17
C12	0	338.73	132.68	2.55
C13	+30	280.80	19.43	14.45

#### ***Effect of machining speed***

**Figure 9:**
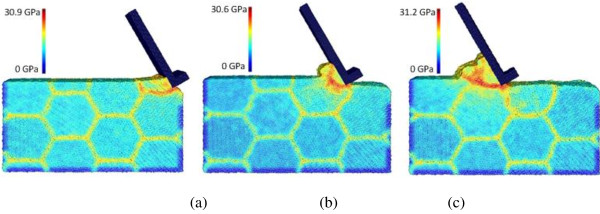
**Chip formations and equivalent stress distributions in nano-scale polycrystalline machining for case C8.** At the tool travel distances of **(a)** 30, **(b)** 120, and **(c)** 240 Å.

**Figure 10:**
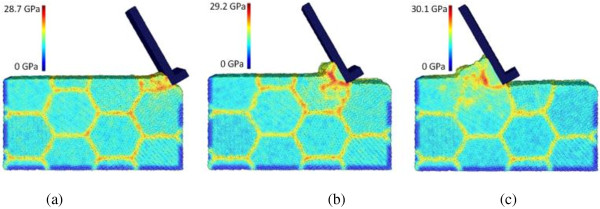
**Chip formations and equivalent stress distributions in nano-scale polycrystalline machining for case C9.** At the tool travel distances of **(a)** 30, **(b)** 120, and **(c)** 240 Å.

The effect of machining speed can be analyzed by comparing cases C4, C8, and C9, which employ the machining speeds of 400, 100, and 25 m/s, respectively. The chip formation and equivalent stress distribution for case C4 is already shown in Figure 3. Figures 9 and 10 depict the results of cases C8 and C9, respectively. The chip morphologies of cases C8 and C9 appear to be quite different from that of case C4. Under the lower machining speeds of 25 and 100 m/s, the chip formation is more like a material pile-up process, and the regular flow of the material along the tool rake face cannot be observed. Also, for these two lower speed cases, the stress concentration along the primary shear zone is more significant than that along the secondary shear zone. Therefore, chip formation seems to be very sensitive to the machining speed for nano-scale polycrystalline machining - the regular uniform chip can only be formed at high machining speeds of more than 100 m/s. In addition, it can be found that lower machining speeds reduce the maximum equivalent stress value. For instance, at the tool travel distance of 240 Å, the maximum equivalent stresses are 42.7, 31.2, and 30.1 GPa at the machining speeds of 400, 100, and 25 m/s, respectively.

**Figure 11:**
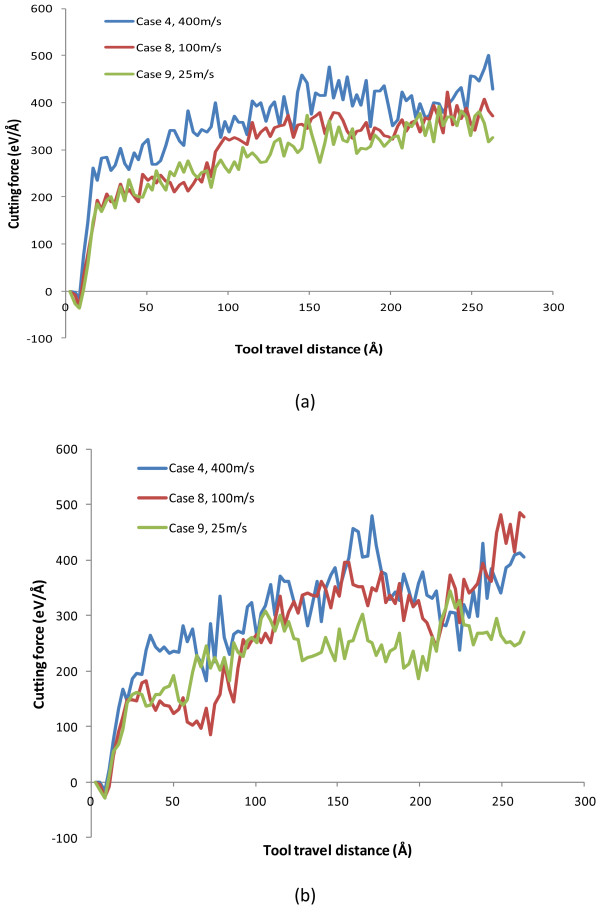
**Evolution of cutting forces at the machining speeds of 25, 100, and 400 m/s. (a)** Tangential force, *F*_*x*_ and **(b)** thrust force, *F*_*y*_.

By comparing the cutting force results shown in Figure 11 and Table 6, it is observed that higher machining speeds constantly introduce higher tangential forces, while the increase of thrust force flats out after the machining speed exceeds 100 m/s. Overall, as the machining speed increases from 25 to 400 m/s, the tangential force increases from 339.85 to 412.16 eV/Å and the thrust force increases from 257.03 to 353.59 eV/Å.

**Table 6:** Average cutting force values with respect to machining speed

**Case number**	**Machining speed (m/s)**	***F***_***x ***_**(eV/Å)**	***F***_***y ***_**(eV/Å)**	***F***_***x***_**/*F***_***y***_
C4	400	412.16	353.59	1.17
C8	100	358.08	355.02	1.01
C9	25	339.85	257.03	1.32

### Effect of grain size

#### ***Cutting force and equivalent stress distribution***

**Figure 12:**
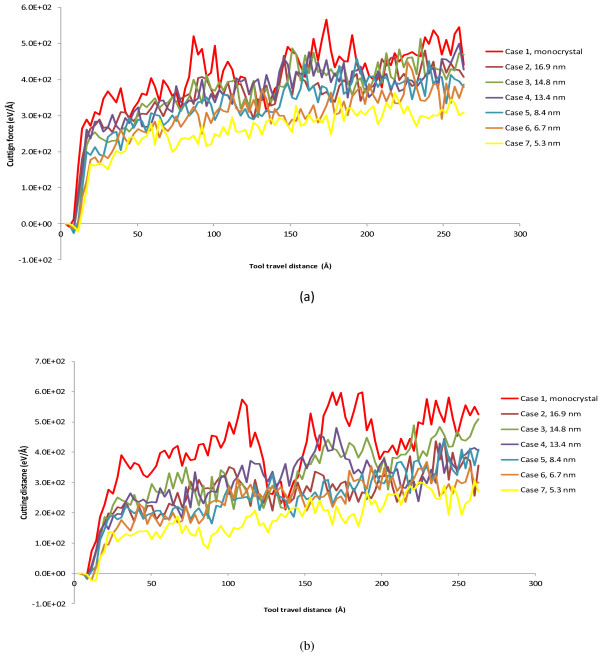
**Cutting force evolution in machining polycrystalline coppers of various grain sizes. (a)** Tangential force and **(b)** thrust force.

**Figure 13:**
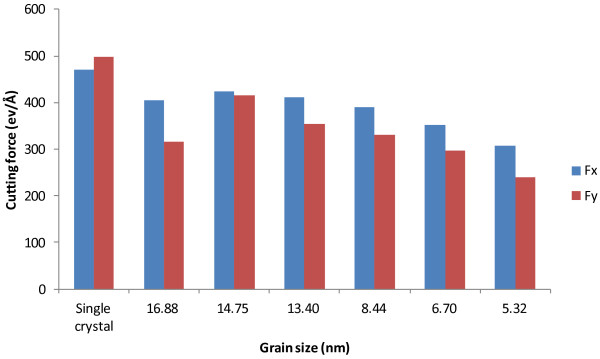
Average tangential and thrust forces for machining polycrystalline coppers of different grain sizes.

**Figure 14:**
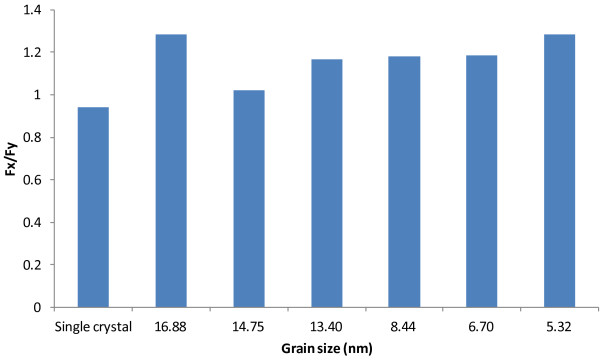
**Ratio of *F***_***x***_**/*F***_***y ***_**for machining polycrystalline coppers of different grain sizes.**

We first investigate the effect of grain size on cutting forces in machining polycrystalline structures. Figure 12 shows the evolution of cutting force components for cases C2 to C7, which represent six polycrystalline structures (i.e., 16.88, 14.75, 13.40, 8.44, 6.70, and 5.32 nm, respectively, in terms of grain size). For benchmarking, the case of monocrystalline machining, namely, case C1, is also added to the comparison. Similarly, the average *F*_*x*_ and *F*_*y*_ values are obtained from the period of tool travel distance of 160 to 280 Å for these cases, and the results are shown in Figures 13 and 14. It is clear that the overall magnitudes of both *F*_*x*_ and *F*_*y*_ for monocrystalline machining are higher than any of the polycrystalline cases. The average *F*_*x*_ and *F*_*y*_ values for case C1 are 470 and 498 eV/Å, respectively. This is reasonable in that the monocrystal copper structure assumes to be perfect without any defects and thus has the highest strength. On the other hand, the existence of grain boundaries, a major form of crystal defects, in all the polycrystalline cases means lower material strengths. Interestingly, the most significant volatility of cutting force is observed in monocrystalline machining. This should be attributed to the highly anisotropic properties of monocrystalline structure and the associated dislocation movement.

More important observations are made with the six polycrystalline cases. It can be seen from Figure 13 that the average cutting forces increase with the increase of grain size in the range of 5.32 to 14.75 nm. In the range, the relative increases are 37.7% and 72.9% for tangential force and thrust force, respectively. However, the cutting forces reverse the increasing trend when the grain size increases to 16.88 nm (case C7). A similar disruption occurs in the trend of *F*_*x*_/*F*_*y*_ with respect to grain size, as shown in Figure 14. The ratio of *F*_*x*_/*F*_*y*_ generally decreases with the increase of grain size, but it rebounds by about 25% when the grain size increases from 14.75 to 16.88 nm. This phenomenon related to grain size and grain boundary is for the first time observed in machining research.

**Figure 15:**
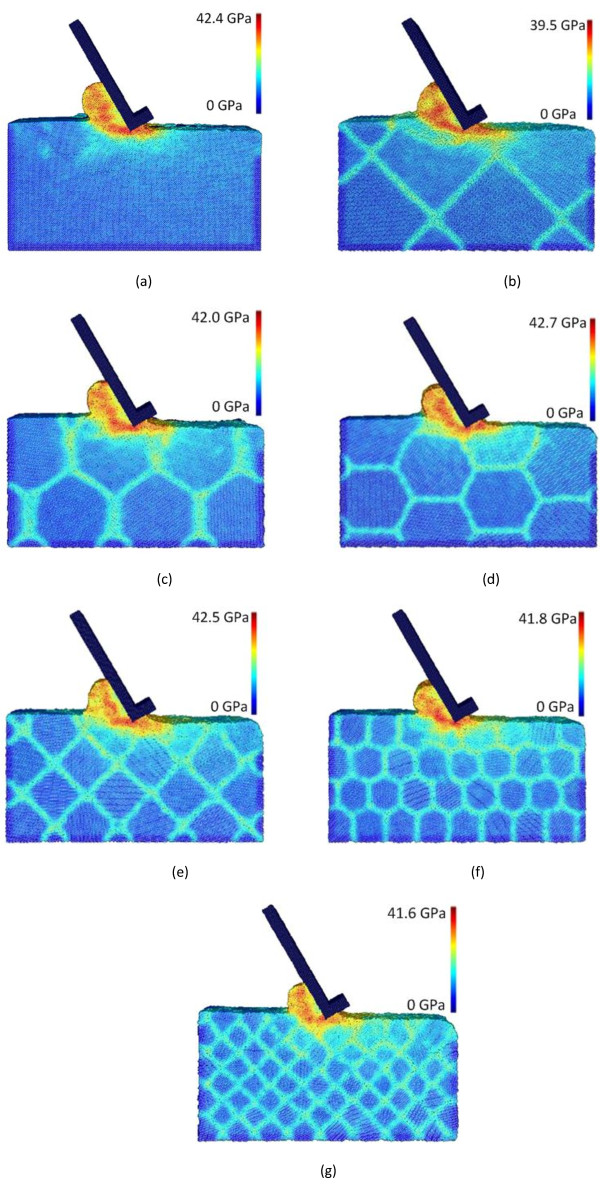
**Equivalent stress distributions in machining polycrystalline coppers with different grain sizes. (a)** Monocrystal, **(b)** 16.88 nm, **(c)** 14.75 nm, **(d)** 13.40 nm, **(e)** 8.44 nm, **(f)** 6.7 nm, and **(g)** 5.32 nm.

Figure 15 depicts the snapshots (tool travel distance = 240 Å) of equivalent stress distribution for the seven polycrystalline cases with various grain sizes (i.e., cases C1 to C7) at the tool travel distance of 240 Å. For each case, the maximum equivalent stress is found to be in the primary shear zone, and it takes the values of 42.4, 39.5, 42.0, 42.7, 42.5, 41.8, and 41.6 GPa for cases C1 to C7, respectively. It overall agrees with the trend of cutting forces, but the magnitude of stress value change is less drastic.

#### ***Inverse Hall–Petch relation***

The influence of grain boundary on material properties can be significant, but it depends on the exact conditions of deformation and the particular material used. In the following, we intend to explain the change of cutting forces with respect to grain size in machining polycrystalline coppers. Usually, the strength of polycrystalline materials is expected to increase if the grain size decreases. For coarse-grain materials, the grain size effect on flow stress can be captured by the empirical Hall–Petch relation, which suggests that the yield stress increases with decreasing grain size by the following:

(6) $$\sigma =\sigma_{0}+K/\sqrt{d},$$

where *σ*_0_ is the yield stress, *K* is the Hall–Petch slope, and *d* is the grain size. According to this classical relation, the force components in machining polycrystalline copper should increase with the decrease of grain size. Indeed, it is the case when the grain size decreases from 16.88 to 14.75 nm. The tangential force increases by 4.6%, and the thrust force increases by 31.6%. However, the Hall–Petch relation is apparently not applicable for polycrystalline machining with grain sizes of 5.32 to 14.75 nm (i.e., cases C2 to C6), in which the cutting forces decrease with the decrease of grain size.

In recent years, it has been discovered that when the grain size of nano-structured materials is smaller than a critical value, the Hall–Petch relation could be inversed [37-39]. In other words, as the fraction of grain boundary atoms increases to a significant level, work softening will become dominant. The inverse Hall–Petch relation indicates that a smaller grain size increases the volume fraction of grain boundary, which facilitates the activation of other deformation mechanisms such as grain boundary sliding and thereby lowers material strength. The inverse Hall–Petch relation indeed matches up with our observation of nano-scale polycrystalline machining in the particular grain size range. Apparently, the decrease in cutting forces with the decrease of grain size is the result of yield strength reduction. The decrease in cutting force can also be further explained as strengthening due to dislocation activity below a critical grain size is ceased, and the kick-in of other mechanisms leads to work softening and thus lowers the force required by the tool to remove the material.

**Figure 16:**
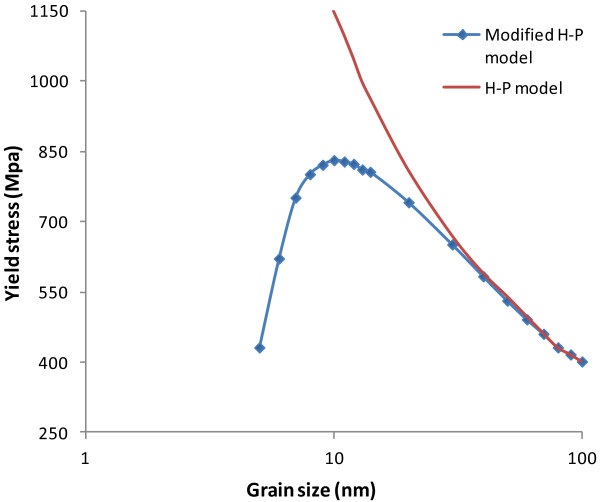
**Predicted yield stress for nano-structured copper as a function of grain size.** Based on the Hall–Petch and modified Hall–Petch relations [40].

In particular, Mohammadabadi and Dehghani developed a modified Hall–Petch equation, which incorporates the negative slope observed between grain size and yield stress [40]. It is in the following form:

(7) $$\sigma =\sigma_{0}+K/\sqrt{d}-\sigma_{\mathrm{in}}f_{\mathrm{gb}},$$

where *σ*_in_ is internal stress along the grain boundary that depends on parameters such as grain boundary thickness, lattice distortions, and grain size, and *f*_gb_ is the volume fraction of the grain boundary. Figure 16 shows the yield stress of polycrystalline copper as a function of grain size under both the conventional Hall–Petch relation and the modified Hall–Petch relation. It can be seen that if the conventional Hall–Petch relation is followed, the yield stress should increase exponentially with grain size reduction. However, the modified Hall–Petch relation indicates that with the decrease of grain size, the yield stress grows at a slower pace to its peak position when the grain size is around 14 nm, and then it starts to drop if the grain size is below this critical value. Note that there are also other literature reporting that for some metals, the critical grain size for the inverse Hall–Petch to take over is about 10 to 15 nm [38,41-43].

A few other studies also show that the flow stress of ultrafine nano-structured materials can decrease as a result of grain size reduction. With the inverse Hall–Petch effect, the deformation is no longer dominated by dislocation motion, while atomic sliding in grain boundaries starts to play the major role [44]. Narayan experimentally studied this phenomenon by pulsed laser deposition to produce nano-crystalline materials [45]. It was discovered that when the copper nano-crystal is less than 10 nm, material hardness decreases with the decrease of grain size. The decrease in the slope of the Hall–Petch curve and eventually the decrease in hardness below a certain grain size can be explained by a model of grain-boundary sliding [46]. Because of this, as the grain size decreases from 61 to 30 nm, the overall material strength increases, but further decrease in the grain size may result in a decrease of strength. The grain-boundary sliding theory is supported by other researchers [47,48], where the small and independent slip events in the grain boundary are seen in the uniaxial tension deformation process of fcc metal with a very small grain size (less than 12 nm).

As such, the modified Hall–Petch relation explains well our discoveries in Figure 13. First, the cutting force increase due to the increase of grain size takes place in polycrystalline machining for the grain size range of 5.32 to 14.75 nm. This is in general consistent with the range reported in the literature that the inverse Hall–Petch effect is dominant. Second, the cutting forces decrease when the grain size becomes larger than 14.75 nm. This is exactly where the regular Hall–Petch effect starts to take over. Therefore, in polycrystalline machining, the critical grain size that divides the regular Hall–Petch and inverse Hall–Petch effects is overall consistent with the critical grain size for yield stress in the literature.

It should also be noted that the maximum equivalent stress in our model is always more than an order of magnitude higher than the yield stress presented in the modified Hall–Petch curve in Figure 16. The huge difference can be attributed to two major factors. First of all, the yield stress data in Figure 16 were obtained from experimental measurements on realistic coppers which actually carry extra defects such as voids and substitutes, while the MD simulation assumes perfect crystalline defect-free copper within each grain. In this case, the material strength of the defect-free copper should be much higher. The literature estimates the theoretical yield stress of copper to be within the range of 2 to 10 GPa [49]. More importantly, much higher stresses are observed in MD simulation of machining because of the strain rate effect. It is well known that the flow stress increases with the increase of strain rate [50]. Machining processes always produce extremely high strain rates in the primary and secondary shear zones, much higher than many other manufacturing processes or regular material property tests. For instance, in the case of machining of AISI 1045 steel at 400 m/min, the maximum strain rate is close to 20,000 s^-1^[34]. On the other hand, the strain rates in material property tests are usually less than 1 s^-1^. For instance, as the strain rate increases from 10^-4^ to 10^4^ s^-1^, the flow stress of oxygen-free high-conductivity (OCHC) copper increases from 0.8 to 1.5 GPa [51], and the yield stress of tantalum increases from 180 to 700 MPa [52]. Moreover, material flow stress increases even more significantly when the strain rate becomes higher than 10^4^ orders of magnitude. Armstrong et al. [53] indicated that the flow stresses of α-Fe at strain rates of 10^4^ and 10^6^ s^-1^ are 800 MPa and 7GPa, respectively. Swegle and Grady [54] showed that for oxygen-free electronic (OFE) copper, the flow stresses are 200 MPa and 2.8 GPa at strain rates of 10^4^ and 10^7^ s^-1^, respectively. The strain rates of the simulated nano-scale machining should be at least 10^8^ s^-1^ because it is proportional to machining speed and inversely proportional to chip thickness. This is partially verified by comparing the maximum stress of 43.6 GPa in case C11 (400 m/s machining speed) with that of 30.1 GPa in case C9 (25 m/s machining speed). Based on these two reasons, it is reasonable that the equivalent stress in this MD simulation study is significantly greater than the yield stress shown in the modified Hall–Petch curve.

#### ***Grain boundary and dislocation interaction***

**Figure 17:**
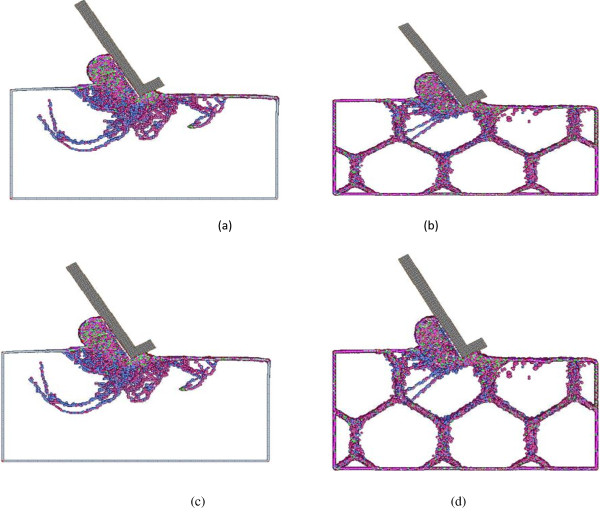
**Dislocation development in polycrystalline machining for simulation cases with different grain sizes. (a)** Monocrystal, **(b)** 14.75 nm, **(c)** 13.40 nm, and **(d)** 5.32 nm.

Figure 17 presents the interaction between grain boundary and dislocation movement inside the work material for the monocrystal case (case C1) and three polycrystalline cases (cases C3, C4, and C7) with a grain size of 14.75, 13.40, and 5.32 nm, respectively. The results are plotted to visualize the changes to the crystalline order of perfect fcc copper. Only defect-related atoms, namely, grain boundary atoms and dislocation atoms, are shown. It can be observed that for the monocrystal copper, the dislocation loops originate from the tool/work interface and/or as-machined surface. The directions of dislocation loops are multiple. It could either propagate along the machining direction beneath the machined surface or penetrate much deeper into the bulk material. Compared with the polycrystalline cases, the dislocation movement in the monocrystal copper is more significant and has greater penetration depth than any of the polycrystalline cases. The cutting force comparison shown above confirms the more drastic dislocation movement that exists in machining monocrystal copper.

As shown in Figure 17b for case C3, since the atomic mismatch between different grains creates a stress field to oppose continued dislocation motion, the dislocations inside grains are clearly blocked by the grain boundary. Therefore, the ‘pile-up’ phenomenon of dislocation occurs as a cluster of dislocations that are not able to move across the grain boundary. The pile-up phenomenon of dislocations is the hallmark mechanism of the normal Hall–Petch relationship. Due to the resistance effect of the grain boundary to the propagation of dislocation, more force needs be applied to move the dislocations across a grain boundary and hence the increase of yield strength and cutting forces. If the grain size continues to decrease, it falls into the inverse Hall–Petch region, as shown in Figure 17c. In this case, the amount of dislocation movement substantially decreases. This indicates that as the grain size drops below the grain boundary strengthening limit, a smaller grain size would suppress the formation of dislocation pile-ups and instead promotes more grain boundary diffusion and sliding, which resolves the applied stress and in turn reduces the material's yield strength. The grain boundary movement for case C7 can be observed from Figure 17d. The shape of many grains becomes irregular, and the grain boundaries beneath the machined surface slide in response to the exerted cutting forces.

## Conclusions

This paper represents an extensive study of using MD simulation approach to investigate machining of polycrystalline structures at nano-scale. It focuses on two important aspects. One is how machining parameters affect the performance of polycrystalline machining. The other is the influence of grain size of polycrystalline copper structures. For this purpose, we generate 13 simulation cases which cover six levels of grain size, namely, 5.32, 6.70, 8.44, 13.40, 14.75, and 16.88 nm; three levels of machining speed; three levels of depth of cut; and three levels of tool rake angle. The results are analyzed based on cutting forces, stress distribution, chip formation, and dislocation development. The major findings are summarized below:

1. Both the tangential and thrust forces increase with the increase of depth of cut for nano-scale polycrystalline machining. The relative increases are 100% and 127% for the tangential and thrust forces, respectively, as the depth of cut increases from 10 to 20 Å. Meanwhile, the maximum equivalent stress value also increases with the depth of cut, but the magnitude of change is much less significant compared with cutting forces.

2. Tool rake angle has a significant effect on machining performances in nano-scale polycrystalline machining. As the tool rake angle changes from -30° to +30°, the tangential and thrust forces decrease by 47% and 1,660%, respectively. The thrust force is much more sensitive to the change of rake angle. The use of nonnegative rake angles reduces the stress concentration in the formed chips.

3. The increase of machining speed generally requires higher cutting forces. In the study, the tangential force increases from 339.85 to 412.16 eV/Å and the thrust force increases from 257.03 to 353.59 eV/Å when the machining speed increases from 25 to 400 m/s.

4. Thanks to the defect-free lattice structure of monocrystal copper, the cutting forces required are significantly higher for the monocrystalline case compared with all polycrystalline cases investigated.

5. Both the regular Hall–Petch relation and the inverse Hall–Petch relation are discovered in investigating the grain size effect in nano-scale polycrystalline machining. In the grain size range of 5.32 to 14.75 nm, the cutting forces increase with the increase of grain size. When the grain size exceeds 14.75 nm, the cutting forces reverse the increasing trend.

6. The mechanisms of Hall–Petch and inverse Hall–Petch effects are discussed. The dislocation-grain boundary interaction shows that the resistance of grain boundary to dislocation movement is the fundamental mechanism of the Hall–Petch relation, while grain boundary diffusion and movement is the reason of the inverse Hall–Petch relation.

## Competing interests

The authors declare that they have no competing interests.

## Authors’ contributions

Dr. JS conceived of the study and developed the framework of simulation models. Mr. YW carried out the molecular dynamics simulation. Dr. XY provided valuable inputs on the discussion and analysis of results. The first and second authors analyzed the results and drafted the manuscript. All authors read and approved the final manuscript.
